# Polyphenols: Modulators of Platelet Function and Platelet Microparticle Generation?

**DOI:** 10.3390/ijms21010146

**Published:** 2019-12-24

**Authors:** Borkwei Ed Nignpense, Kenneth A. Chinkwo, Christopher L. Blanchard, Abishek B. Santhakumar

**Affiliations:** 1 School of Biomedical Sciences, Charles Sturt University, Locked Bag 588, Wagga Wagga, NSW 2678, Australia; bednignpense@csu.edu.au (B.E.N.); kchinkwo@csu.edu.au (K.A.C.); cblanchard@csu.edu.au (C.L.B.); 2 Australian Research Council (ARC), Industrial Transformation Training Centre (ITTC) for Functional Grains, Graham Centre for Agricultural Innovation, Charles Sturt University, Wagga Wagga, NSW 2650, Australia

**Keywords:** platelets, polyphenols, platelet microparticles, aggregation, flow cytometry, atherosclerosis

## Abstract

Platelets and platelet microparticles (PMPs) play a key role in the pathophysiology of vascular disorders such as coronary artery disease and stroke. In atherosclerosis, for example, the disruption of the plaque exposes endogenous agonists such as collagen, which activates platelets. Platelet hyper-activation and the high levels of PMPs generated in such situations pose a thrombotic risk that can lead to strokes or myocardial infarctions. Interestingly, dietary polyphenols are gaining much attention due to their potential to mimic the antiplatelet activity of treatment drugs such as aspirin and clopidogrel that target the glycoprotein VI (GPVI)–collagen and cyclooxygenease-1 (COX-1)–thromboxane platelet activation pathways respectively. Platelet function tests such as aggregometry and flow cytometry used to monitor the efficacy of antiplatelet drugs can also be used to assess the antiplatelet potential of dietary polyphenols. Despite the low bioavailability of polyphenols, several in vitro and dietary intervention studies have reported antiplatelet effects of polyphenols. This review presents a summary of platelet function in terms of aggregation, secretion, activation marker expression, and PMP release. Furthermore, the review will critically evaluate studies demonstrating the impact of polyphenols on aggregation and PMP release.

## 1. Introduction

In Australia, despite decreasing deaths and hospitalization rates, cardiovascular diseases remain a major disease burden [1]. According to the World Health Organization, in 2016 cardiovascular diseases accounted for an estimated 31% of deaths worldwide, with 85% of all the deaths being a result of a stroke or myocardial infarction [2]. Platelets are anucleate fragments of a larger cell—the megakaryocyte—that play a major role in the pathophysiology of such conditions [3]. Structurally, however, they are similar to most cells in that they have receptors that respond to extracellular signals, contain mitochondria and other organelles, and release bioactive compounds [3]. They contain lysosomes, alpha granules, and dense granules. During vascular damage platelets adhere to the damaged area, are activated and release contents from both alpha and dense granules to facilitate normal physiological responses including haemostasis, inflammation, and wound healing [4]. However, in an arteriosclerotic environment, a rupture in the plaque may activate platelets, resulting in clot formation in the narrowed blood vessel, consequently leading to a stroke or myocardial infarction. Platelet hyper-activation associated with such cases is considered a risk factor and can be evaluated by measuring the markers or products of platelet activation.

Activated platelets express receptors and secrete granular contents such as von Willebrand factor (vWF), P-selectin, and ADP, which facilitate aggregation [4]. Macrovesicles, also known as platelet microparticles (PMPs), are released upon activation and are involved in platelet function [4,5,6]. Aggregometry and flow cytometry are common platelet function tests used to measure platelet aggregation and activation biomarkers, respectively [7]. In clinical settings, antiplatelet drugs, such as aspirin and clopidogrel, used in the treatment of cardiovascular conditions can be monitored using these two common methods [8]. The drugs aspirin and clopidogrel antagonize the cyclooxygenease-1 (COX-1) enzyme and P2Y_1_/P2Y_12_ platelet receptor to reduce platelet hyper-activation. P2Y_1_/P2Y_12_ is a receptor that binds to ADP to trigger platelet activation. Clopidogrel, due to its selectivity for the P2Y_1_/P2Y_12_, is able to reduce ADP-induced aggregation and activation-dependent receptor expression and secretion [9,10]. On the other hand, aspirin inhibits COX-1—a platelet intracellular enzyme responsible for catalyzing the conversion of arachidonic acid to thromboxane A2 (an agonist which enhances activation and aggregation) [11]. However, due to COX-1 being a common signalling molecule in multiple pathways of platelet activation, aspirin is able to inhibit activation and aggregation induced by multiple agonists [12,13]. Furthermore, although these drugs are commonly used and effective in many cases, unresponsiveness and side effects have been associated with their administration [14].

Fortunately, diets rich in plant-based compounds such as polyphenols have displayed cardiovascular health benefits via the inhibition of platelet function. Several studies have demonstrated that polyphenols may affect platelet activation, adhesion, degranulation, and aggregation by targeting specific thrombogenic pathways, for example, P2Y_1_/P2Y_12_–ADP, glycoprotein VI (GPVI)–collagen, protease-activated receptor 1 (PAR1)–thrombin, and the COX-1–thromboxane pathways. In vitro studies have evaluated the inhibitory effect of polyphenols on each of these activation pathways using different types of aggregometry and flow cytometry-based activation assays. However, there is still a limited understanding of the interaction between polyphenols and PMP generation. The methods used in many of these studies requires careful evaluation, especially considering the relatively low bioavailability of polyphenols. This review will provide an overview of the physiological role of platelet function in haemostasis, the agonist-induced pathways involved, the release of PMPs, and the platelet function tests used. Furthermore, studies investigating the antiplatelet therapeutic potential of dietary polyphenols will be evaluated with a specific focus on the impact on aggregation and PMP generation.

## 2. Platelet Activation and Platelet Microparticle Production

### 2.1. Introduction to Primary Haemostasis

Primary haemostasis, which is essentially platelet aggregation and the formation of a platelet plug, is mediated by cell receptor interactions. Under normal circumstances, the endothelial lining of blood vessels does not adhere to circulating platelets. However, in situations where the blood vessel wall is damaged due to trauma or atherosclerotic plaque disruption, the platelets adhere to the vessel wall, leading to receptor activation, conformational change, degranulation, and aggregation [11]. This platelet adhesion is facilitated by vWF forming a bridge between the glycoprotein (GP) IIb/IIIa (also referred to as integrin α_IIb_β_3_ or CD41/CD61) receptor and exposed collagen of the vessel wall [15]. The GP IIb/IIIa receptor can also bind directly to exposed collagen or fibrinogen to activate platelets. The activated platelets release granule contents that activate other platelets in an autocrine or paracrine fashion. Platelet aggregation is finally achieved by crosslinking of adjacent platelets via fibrinogen or vWF bound to the GP IIb/IIIa receptor.

### 2.2. Platelet Activation via Other Glycoprotein and G-protein Coupled Receptors

Platelets can be activated via other receptors. GPVI is a platelet receptor that binds to collagen with high affinity probably due to its dimer structure clustering upon adhesion [16]. Another glycoprotein, the GP Ib-IX-V complex, can arrest circulating platelets by binding to vWF and promoting platelet adhesion. The GP Ib*α* unit of the complex binds to the A1 domain of vWF and filamin A in the platelet cytoplasm [17]. Other than glycoprotein receptors, purinergic receptors and protease-activated receptors on platelets play a key role in platelet activation. P2Y_1_, P2Y_12_, and P2X_1_ are three distinct purinergic receptors that bind adenine nucleotides to induce activation [18]. The first two are G protein-coupled receptors (GPCRs) and the last is a receptor ligand-gated cation channel [15]. Since P2Y_1_ is responsible for initiating ADP-induced aggregation and P2Y_12_ is necessary for completing the aggregation, co-activation of both receptors is essential for ADP-induced aggregation [18]. Activated purinergic receptors increase the concentration of Ca^2+^ and activate downstream signalling pathways that result in ADP and ATP release. Through a positive feedback loop, adenine nucleotides act on the purinergic receptors to sustain platelet activation or activate neighbouring platelets to promote plug formation [18]. Another class of GPCRs, protease-activated receptors (PARs), are activated by a relatively strong platelet agonist, thrombin, which also functions in secondary haemostasis to stabilize clot formation via the conversion of fibrinogen to fibrin. The PAR family consists of PAR1, PAR2, PAR3, and PAR4 [19,20]. Thrombin induces platelet activation mainly through PAR1 and PAR4, with PAR1 initiating a stronger response than PAR4 at relatively lower thrombin concentrations [15]. Other receptors such as alpha 2-adrenergic receptors and the 5-hydroxytryptamine (5-HT) receptors interact with epinephrine and serotonin respectively to induce platelet activation [21,22].

### 2.3. Downstream Platelet Intracellular Signalling

**Figure 1:**
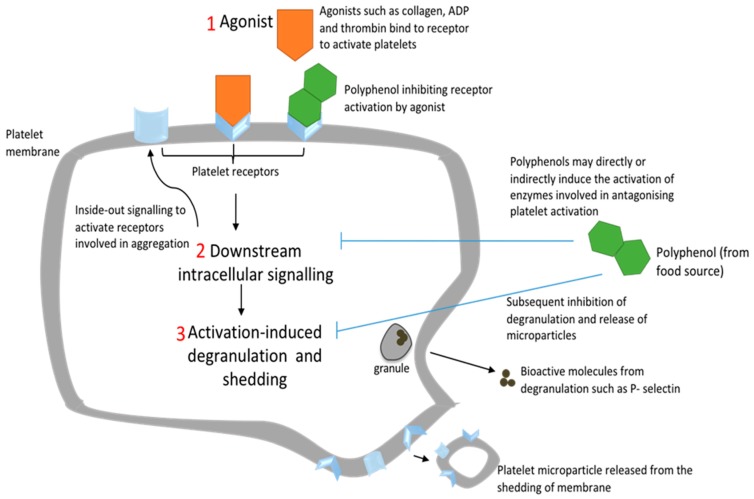
Simplified schematic diagram of proposed inhibitory actions of polyphenols on platelet activation. (**1**) Polyphenols may block receptor–agonist interactions such as P2Y_1_/P2Y_12_–adenosine diphosphate (ADP), glycoprotein VI (GPVI)–collagen, and protease-activated receptor 1 (PAR1)–thrombin. (**2**) Downstream activation signalling is inhibited by the decreased receptor action, scavenging of free radicals, or by polyphenols directly interacting with enzymes such as cyclooxygenease-1 (COX-1), phospholipase C (PLC), and protein kinase C (PKC). Decreased intracellular signalling results in decreased Ca^2+^ release and expression of receptors such as GPIIa/IIIb necessary for forming platelet aggregates. (**3**) The inhibition of activation by polyphenols results in a reduction in degranulation and platelet microparticle (PMP) release.

All of the already mentioned receptors, whether of the GPCR or glycoprotein types, result in individual downstream signalling pathways that may converge and lead to a conformational change and degranulation upon platelet activation by an agonist [23]. Each pathway involves key enzymes that catalyze the process. One such enzyme is the cyclooxygenase-1 (COX-1) enzyme that catalyzes the conversion of arachidonic acid to prostaglandin G2 and prostaglandin H2 [24]. The latter is converted to thromboxane A2 (TxA_2_), a potent vasoconstrictor and agonist. TxA_2_ binds to its corresponding thromboxane receptor to induce platelet activation [24]. The complex signalling results in conformational change and integrin activation. Some key mediators involved in signalling include phospholipase C (PLC), protein kinase C (PKC), and phosphatidylinositide-3-kinase (PI3K) [23]. Upon activation of a GPCR, PLC and P13K enzymes are activated [23]. PLC catalyzes the conversion of phosphatidylinositol 4,5-bisphosphate (PIP2) into diacylglycerol (DAG) and inositol 1,4,5-trisphosphate (IP_3_), whereas P13K activation results in Ca^2+^ release from the dense tubules [22]. DAG also activates PKC and IP_3_ receptors, which allow the release of Ca^2+^ from the tubules [23]. PKC consists of isoforms that have a broad role in TxA_2_ synthesis, integrin activation, conformational change, and degranulation [23]. Overall, platelet conformational change is mediated by GPCRs that elevate Ca^2+^ which couples to Rho GTPases [23]. Rho mediates the overall platelet morphological change by activating myosin light chain kinase [23]. The rise in intracellular Ca^2+^ also leads to the activation of integrin α_IIb_β_3_ by modulating GTPase Rap 1 activation [25] (Figure 1). The integrin activation involves a change in the ligand-binding site of the extracellular domain that results in the switch from a low-affinity bent conformation to a high-affinity extended conformation. Finally, granule secretion is modulated by intracellular Ca^2+^ and PKC activation [23]. This activation-induced exocytosis also results in the release of microparticles.

### 2.4. Platelet Microparticles (PMPs)

Microparticles are released from all blood cells, however, PMPs make up to 60%–90% of all microparticles in circulation [5,26]. PMPs are like other extracellular vesicles such as exosomes and apoptotic bodies. However, they are generally bigger than exosomes, having a size range of 100–1000 nm, whereas exosomes are about 30–100 nm. Apoptotic bodies, on the other hand, are larger than both vesicles, with an average size greater than 1 μm [5]. Whereas exosomes are released from intracellular multivesicular bodies, PMPs are released from the exocytotic budding of the cell membrane [5]. The mechanism for PMP generation is not fully understood, however, three possible mechanisms have been identified: GPIIb/IIIa signalling, shear stress-induced, or agonist-induced platelet activation [5]. Resting platelets can shed PMPs due to a destabilization of the actin cytoskeleton in response to GPIIb/IIIa signalling in the absence of an agonist [5]. PMPs can also be generated in response to shear stress—frictional forces caused by the blood flow and pressure in the vasculature [27]. In response to agonist-induced platelet activation, an elevation of intracellular Ca^2+^ may initiate the loss of lipid asymmetry and hence cytoskeletal integrity leading to the shedding of PMPs [5] (Figure 1). In terms of clearance of PMPs, the half-life is 30 min and 10 min in mice and rabbits respectively [5]. However, in contrast to platelets having a half-life of about 10 days, the half-life of PMPs in humans is not known [5]. The plasma membrane of PMPs exposes negatively charged phospholipids, such as phosphatidylserine, that are involved in phosphatidylserine-dependent phagocytosis of PMPs [5]. Many of the same receptors and bioactive molecules in platelets are also found in PMPs. Receptors such as GPIIb/IIIa, P-selectin, and vWF involved in primary homeostasis can be expressed on the membrane [5]. Procoagulant factors, nucleic acid, mitochondria, chemokines, and cytokines found in a PMP allow it to influence pathways in inflammation, atherosclerosis, and thrombosis [26]. Interestingly, Dean et al. [28] demonstrated through proteomic and functional analysis that PMPs can be classified in terms of size and bioactive contents. This may imply that different PMPs have different functions in the blood—an area to be explored further in future research.

## 3. Platelet Function Tests

### 3.1. Aggregometry

Two common in vitro methods used for measuring platelet aggregation are whole blood impedance and light transmission aggregometry (LTA) [29]. Both tests are based on the principle that platelets aggregate after activation. In terms of light transmission aggregometry, platelet-rich plasma (PRP) is acquired by washing or gel filtration of platelets [29]. Agonist-induced aggregation is then quantified by measuring the transmission of light through the sample of platelets [30]. On the other hand, the impedance method primarily uses whole blood to induce platelet aggregation [29]. Upon incubating and stirring of the blood, platelets activate and stick to the surface of two electrodes, thereby increasing electrical resistance between them [30]. This technique has an advantage over the LTA method due to the fact that blood samples do not need tedious processing before platelet activation consequently avoiding artefactual platelet activation [30].

The lumi-aggregometry is a bioluminescent assay that works on the principle that activated platelets release ADP and ATP from their dense granules. Alongside an agonist, a luciferin-luciferase reagent is added which reacts with ATP (converted from ADP) to form luciferyl adenylate [29,31]. Luciferyl adenylate undergoes oxidization resulting in a chemiluminescent reaction where light is emitted and quantified by the analyzer relative to an ATP standard [29]. However, this method allows the simultaneous measurement of whole blood or PRP aggregation and ATP released [30]. The method is also poorly standardized resulting in high inter- and intra- variability for ATP release stimulated by different agonists [29,31].

Other aggregometry techniques include point-of-care tests such as the platelet function analyzer (PFA), which is shear stress-based, and Verifyow based on turbidimetric-optical detection. Both tests rapidly detect aggregation in whole blood and are commonly used to monitor antiplatelet therapy [32]. However, point-of-care tests are less used in in vitro antiplatelet research. Moreover, because in vitro studies poorly reflect in vivo situations, a few in vivo platelet function tests in mouse models have been developed. These take into consideration more mediators of platelet function in the vasculature environment [33,34,35].

### 3.2. Flow Cytometry

Flow cytometry is another common technique used in platelet activation studies. The technique involves the qualitative and quantitative measurement of cells [30]. Cells are differentiated based on size (forward scatter), internal complexity (side scatter) and surface receptor expression [30,36]. The instrument’s laser allows for optical and fluorescence evaluation of antibody-labelled cells [36]. The intensity of light emitted is proportional to the number of antibodies attached to the cells [37]. Due to the varying types of receptors on platelets, an array of fluorescent antibodies can be used for labelling [21]. Receptors expressed in an activated platelet can be labelled using fluorescent conjugated antibodies that serve as activation markers. For example, the CD62P antibody binds to P-selectin and PAC-1 binds to GPIIb/IIIa in activated platelets [38]. This flow cytometric analysis can be performed using platelets in whole blood, platelet-rich plasma, and even in platelet-poor plasma rich in PMPs.

The principle of PMP measurement works similarly to platelet activation analysis [39,40]. Most of the same fluorescent antibodies such as PAC1, P-selectin, and CD41 are used for differentiating resting platelets from activated platelets and PMPs. Thus, forward scatter analysis is used to detect PMPs as they are much smaller than normal platelets. However, to prevent counting small platelets or debris as PMPs, gating should be carefully adjusted. Silica beads, for example, can be used to establish a gating range fitting for PMPs [39]. Besides, careful consideration needs to be given to pre-analytical errors during sample collection and processing.

### 3.3. Other Platelet Function Tests

Other platelet function tests for PMP analysis include imaging flow cytometry, which is more sensitive in detecting smaller PMPs and differentiating them from debris by acquiring a high-resolution image of a single particle [41]. The electron microscopy and cryo-microscopy techniques can identify smaller sized PMPs and provide morphological information [39]. The enzyme-linked immunoassay (ELISA) method can also be used to count and perform functional analysis on PMPs but without size information [39]. In addition, the vasodilator-stimulated phosphoprotein (VASP) phosphorylation assay is a special flow cytometry method that has been used in research studies to evaluate platelet function [42,43,44]. It works on the principle that activation of the P2Y_12_ receptor by ADP reduces VASP phosphorylation and thus inhibition of receptor activation increases VASP phosphorylation. Increased VASP phosphorylation correlates with reduced αIIbβ3 activation and platelet aggregation—measured as a platelet reactivity index. Other platelet function tests include screening tests such as bleeding time, evaluation of the entire clot development such as in thromboelastography, and the evaluation of thromboxane metabolites using radio- or enzyme- linked assays [45]. All the above platelet function tests are currently used in the monitoring of antiplatelet therapy and diagnosis of platelet disorders but may prove beneficial in polyphenol research—evaluating the health properties of food.

## 4. Role of Polyphenols in Modulating Platelet Aggregation and PMP Production

### 4.1. Polyphenols

Polyphenols are natural bioactive compounds that are present in plants. They are found in a variety of food sources including fruits, chocolate, beverages, vegetables and grains [46]. The basic structure of all polyphenols consists of an aromatic ring with a hydroxyl substituent. Although numerous classes exist based on carbon skeleton structure, the number of phenol rings and structural elements, polyphenols can be classified into two main groups: flavonoids and nonflavonoids [46,47]. Flavonoids are further classified into flavonols, isoflavones, flavanones, anthocyanins, and flav-3-ols, whereas nonflavonoids consist of phenolic acids, hydrolysable tannins, and stilbenes. The polyphenol content in foods differs, with fruits having more content than some legumes and cereals [46]. Only a small proportion of dietary polyphenols ingested are absorbed by the small intestine. The remainder is transformed by gut microbiota and then finally excreted via the kidneys [48]. The biotransformation that occurs involves hydrolysis of the glycone (polyphenol bound to a sugar moiety) and conjugation of the aglycone (polyphenol without the sugar moiety [49]. The conjugation process involves enzymatic processes, including methylation, sulfation, and glucuronidation, that occur to detoxify compounds and facilitate the elimination from the body [50]. The polarity, functional groups, and overall structure of polyphenols allow them to induce favourable biological responses via free radical scavenging, metal chelation, and protein or receptor binding [47,51]. There is evidence that polyphenols, especially flavonoids (due to the functional groups attached to their A, B, and C rings), can induce several health benefits including the potential to alleviate atherosclerosis, thrombosis, and inflammation by modulating platelet function [52,53,54,55].

### 4.2. Impact of Polyphenols on Platelet Aggregation

Several studies have investigated the anti-thrombogenic effect of polyphenols by measuring aggregation in whole blood or isolated platelets. Few studies have evaluated ATP release in conjunction with aggregation. In a crossover double-blind study, Singh et al. [56] demonstrated the effect of exercise and training on platelet aggregation, as well as ATP release in whole blood. Cocoa polyphenols were used as a treatment to investigate its role in the platelet activation status of participants. Results showed an increase in ATP release post-exercise, independent of whether participants were trained or sedentary. However, the supplementation with cocoa polyphenols did not normalize the high ATP release. Collagen-induced aggregation post-exercise resulted in elevated ATP release without an increase in platelet aggregation. Considering the generation of free radicals can be attributed to strenuous exercise, the increased platelet degranulation post-exercise may be as a result of exercise-induced oxidative stress coupled with collagen activation via the GPVI pathway [11]. However, since there was no change in aggregation, a flow cytometric evaluation of GPIIb/IIIa expression could have been beneficial. Moreover, cocoa is a rich source of flavonoids, which comprise catechins, proanthocyanins, anthocyanins, and flavonol glycosides that may modulate platelet function [57]. Although cocoa supplementation has consistently shown inhibition of platelet activation, especially when consumed on an acute or chronic basis, the study reported no platelet inhibition post-exercise [58]. This may be attributed to low bioavailability of cocoa polyphenols post-intake [57], i.e., peak plasma concentrations of cocoa polyphenols could have been inadequate to inhibit platelet ATP release amplified by exercise-induced oxidative stress.

Another study by Singh et al. [59] described a dose-dependent decrease in platelet aggregation and ATP release when whole blood was incubated with olive leaf extracts. Olive leaves are rich in polyphenols oleuropein (main ingredient), flavones, flavonols, and other phenols [60]. The antioxidant attributes of olive leaf polyphenols were believed to be responsible for the inhibition of oxidative stress-induced platelet activation, as seen previously [56]. Moreover, the polyphenols may synergistically attenuate platelet degranulation by scavenging H_2_O_2_, a substrate involved in the COX-1 pathway of platelet activation. However, a limitation of the study is its small sample size and selection of only males, posing the possibility of gender bias. Nevertheless, other studies have shown that polyphenols may inhibit aggregation and secretion by interacting with downstream intracellular pathways within platelets.

Chlorogenic acid found in fruits such as cherries, apples, and plums has been shown to inhibit collagen and ADP-induced platelet aggregation and ATP release in washed platelets [61]. The study by Fuentes et al. [61] demonstrated dose-dependent inhibition of aggregation and subsequent platelet ATP release by the purinergic and GPVI pathways of activation induced by ADP and GPVI respectively. This correlated with results indicating an increase in platelet cyclic adenosine monophosphate (cAMP) levels and protein kinase A (PKA) activation. The elevation of cAMP levels activates PKA, which is a potent antagonist of platelet activation. An apparent limitation to the study was the small sample size and thus ongoing studies with larger sample sizes should be conducted to validate findings. Nevertheless, another polyphenol ellagic acid has been shown to modulate platelet intracellular signals. Chang et al. [62] demonstrated the inhibitory effect of ellagic acid on ATP release as well as both PLC and PKC activation. However, both studies were conducted in vitro with washed platelets and this poses a challenge with translating results to reflect complex in vivo processes. Nevertheless, the studies may highlight a possible polyphenol-mediated attenuation of aggregation and secretion via the PLC/PKC activation pathways.

Pigmented rice-derived polyphenols have also shown inhibition of platelet ATP secretion. Zhou et al. [63] conducted in vivo trials on mice fed with high-fat diets. The impact of dietary intervention with the anthocyanin cyanidin-3-glucoside (C3G) purified from black rice was investigated. C3G significantly attenuated thrombin-induced platelet activation and ATP secretion in the mice fed with high-fat diets. This observation concurs with a study by Yao et al. [64] that demonstrated that C3G inhibited thrombin and collagen-induced platelet activation. More so, an earlier study conducted in vivo showed that when rabbits were fed with black or red rice, this resulted in an increase in antioxidant level and a decrease in atherosclerotic plaque formation induced by dietary cholesterol [65]. Recent studies have also confirmed antioxidant and anti-inflammatory properties of a variety of pigmented rice rich in the anthocyanin C3G [66,67]. However, the fact that some of these studies were conducted in animal models coupled with the known low bioavailability of anthocyanins poses a challenge to translate results to reflect the human in vivo situation. Other low bioavailable polyphenols found in olive oil have been shown to have antioxidant and antiplatelet activity [68].

A study by Correa et al. [69] compared the anti-aggregation action of virgin olive oil polyphenols with aspirin. Results showed a similar anti-aggregation action between hydroxytyrosol acetate (HT-AC) and aspirin. However, HT-AC showed a higher anti-aggregation effect than its phenolic counterpart hydroxytyrosol (HT). In comparison with PRP, a greater anti-aggregation effect of aspirin and HT-AC was observed in whole blood when platelets were induced with ADP or collagen. This may indicate an alternative aggregation pathway that involves interactions between leucocytes, erythrocytes, platelets, and other plasma proteins. Aspirin and the two phenolic compounds may stimulate leucocyte nitric oxide (NO) production that might contribute to the anti-aggregation effect. According to Banerjee et al. [70], NO, in addition to inhibiting aggregation through the synthesis of cAMP and cyclic guanosine monophosphate (cGMP), can inhibit platelet aggregation through attenuating TxA_2_ synthesis, which is a part of the COX-1 pathway.

Using whole blood impedance, another study evaluated the antiplatelet benefit of polyphenol-rich product propolis. Bojic et al. [71] demonstrated that propolis inhibited ADP-induced platelet aggregation in whole blood. The study measured the anti-aggregatory effect of propolis ethanolic extracts in whole blood. The most potent extracts that inhibited aggregation were rich in flavonoids. As reviewed by Faggio et al. [72], there are numerous pathways of anti-aggregation including inhibition of intracellular signalling enzymes (such as PLC and COX-1) or even reducing the oxidative burst. The inhibition of the COX-1 pathway appears to be the most common. However, with the study by Bojic et al. [71], the inhibition of the P2Y_12_ pathway of activation may result in a decrease in the expression of the GPIIb/IIIa receptor which is involved in crosslinking activated platelets to form platelet aggregates [72]. Overall, however, the rich source of flavonoid and phenolic acids together in propolis may highlight a synergistic anti-aggregatory effect. As per the study by Wright et al. [54], the structural basis of polyphenol inhibition, specifically flavonoids, has been demonstrated in collagen-induced aggregation to be possibly influenced by functional groups within and on the periphery of the core flavonoid skeleton (Figure 1). Bojić et al. [73] showed that in terms of the flavonoid aglycone, increases in the number of hydroxyl groups on the A and B rings may decrease flavonoid activity, whereas the presence of O-methyl groups may increase activity. On the other hand, a hydroxyl group at position C3 can increase flavonoid activity.

### 4.3. Impact of Polyphenols on PMP Production

The impact of polyphenols on platelet activation has been well documented with several reviews. Few studies have investigated platelet activation and PMP enumeration in healthy and unhealthy populations undergoing dietary intervention trials (Table 1). A random crossover trial by Zhang et al. [74] evaluated the difference in PMP generation and surface molecule expression between individuals with or without type 2 diabetes and obesity. Employing 30 subjects, PMPs were isolated from blood by ultracentrifugation and then incubated with antibodies specific for PMPs as well as leucocyte- and monocyte-derived microparticles. The study found that the average PMP in the diabetic subjects was higher than those without diabetes. Also, the amount of fibrinogen-, P-selectin-, and tissue factor-positive PMPs was elevated in type 2 diabetics independent of obesity status. In conjunction with these results, another study conducted by Zhang et al. [75] demonstrated that a healthy chronic diet, particularly one enriched with oats, reduced these specific types of PMPs in subjects with type 2 diabetes.

These PMPs elevated in type 2 diabetics may play a role in inflammation and thrombosis. P-selectin, for example, is released from the alpha granule upon platelet activation and possesses cell surface adhesion properties. P-selectin-positive PMPs may aid in the recruitment and adhesion of leucocytes and platelets during the early development of atherosclerosis. In addition, fibrinogen is known to be involved in the coagulating cascade and platelet plug formation. Hence, fibrinogen-positive PMPs may mediate thrombosis formation or aid inflammation in type 2 diabetics. As tissue factor is an activator in the extrinsic pathway of the coagulation cascade, it may also play a role in the procoagulant properties of PMPs [75]. Furthermore, oats are a rich source of polyphenols. A phenolic alkaloid, avenanthramide, found mainly in oats, has demonstrated anti-inflammatory and antioxidant activity [76,77,78]. Avenanthramides may play a role in reducing the surface marker-specific PMPs by either directly attenuating platelet activation by interacting with activation receptors or indirectly by scavenging free radicals involved in oxidative stress-induced activation. However, overall, the mechanism of the specific types of PMPs in the development of atherosclerosis or inflammation in type 2 diabetes is not known and it is therefore difficult to highlight a specific pathway for inhibition of platelet PMP formation. In vitro investigation of the antiplatelet effect of avenanthramides may elucidate this issue.

Gallic acid is a polyphenol that has shown positive effects in obese and overweight subjects. For example, pomegranate, a rich source of gallic acid and ellagic acid, has been demonstrated to have anti-inflammatory effects and to significantly decrease LDL cholesterol but significantly increase HDL cholesterol in obese and overweight subjects [46,76,79]. Nevertheless, Chang et al. [80] have shown that gallic acid inhibits ADP-induced platelet aggregation by reducing intracellular Ca^2+^ concentration, attenuating phosphorylation-based transduction pathways and reducing P-selectin secretion. Another study by Olas et al. [81] found out that grape seed extracts (containing a mixture of polyphenols including gallic acid, falvan-3-ols, and proanthocyanins) inhibited thrombin- and thrombin receptor-activation peptide (TRAP)-induced platelet activation and PMP formation more than another polyphenol, resveratrol [81,82,83]. Both polyphenol treatments may inhibit PAR receptor activation by modulating its response to proteolytic (thrombin) and non-proteolytic (TRAP) activation. Furthermore, resveratrol and its analogue isorhapontigenin have been demonstrated to inhibit ADP induced platelet activation [84,85]. However, grape seed extracts contain multiple polyphenols that may interact with several pathways in platelet activation, leading to decreased degranulation and PMP shedding, thus contributing to its higher inhibition than pure resveratrol. In addition, due to the antioxidant properties of grape seed extracts, its polyphenols may alleviate the oxidative stress-induced platelet activation and PMP shedding by scavenging free radicals [81].

In vitro studies by López Andrés et al. [86] investigated the effect of red wine polyphenols on PMP generation in aldosterone-salt-mediated hypertension. About 149 uniephrectomized male Sprague–Dawley rats were treated with aldosterone-salt to induce hypertension and then treated for a month with either spironolactone (a mineralocorticoid receptor antagonist) or Provinols™ to monitor the effect on inflammation, oxidative stress, endothelial dysfunction, and PMPs and endothelial microparticles (EMPs). Provinols™, composed of red wine polyphenols such as proanthocyanins, catechins, flavonols, and tannins, were found to prevent elevation of circulating microparticles, inflammation, oxidative stress, and endothelial dysfunction. In comparison, spironolactone had a similar effect but reduced blood pressure and aldosterone-induced PMP generation via shear stress. In conjunction with these results, Lu et al. [87] found a positive correlation between circulating microparticles (PMP and EMPs) and blood pressure, as well as a positive correlation between platelet activation markers (P-selectin and vWF) and circulating microparticles in patients with chronic kidney disease. Based on both studies, it can be speculated that polyphenols may diminish the development of endothelial impairment by modulating the vascular environment and interfering with platelet activation pathways that lead to PMP generation. More so, polyphenols may alter shear stress indirectly due to their modulation of blood flow via NO release and endothelium relaxation [27]. Shear stress has been shown to be physiologically important for PMP shedding [88].

Giacomazzi et al. [88] reported that agonist-induced PMP generation without shear stress conditions is significantly lower than with shear stress. Collagen and thrombin were the strongest agonists for PMP generation. The study highlighted that PMP generation via the GPIIb/IIIa and GPVI intracellular activation pathways may be enhanced by secretion of endogenous agonist ADP and TxA_2_ in such a way that antiplatelet agents that antagonize the respective P2Y_12_ and thromboxane receptors attenuate PMP generation. Therefore, polyphenols that inhibit platelet activation via the COX-1 and P2Y_12_ pathway may also attenuate PMP generation in activated platelets [89,90]. Further studies are needed to investigate the possible interactions of dietary polyphenols in PMP generation pathways as a result of in vitro agonist induction and in vivo pathophysiological mechanisms in diseased populations.

## 5. Evaluation of Methods Used for Polyphenol Antiplatelet Studies

When investigating the antiplatelet properties of polyphenols, the study design is pivotal in addressing the hypothesis. Many studies have used in vitro methods, with a number of these being small sample sizes. Though such in vitro analysis is instrumental in the deduction of structure-activity relationship of polyphenol extracts, they do not take into consideration the extensive chemical transformation that polyphenols undergo during absorption. The metabolites, rather than the parent compounds ingested, may be responsible for antiplatelet activity in vivo hence the need for a pipeline of studies (in vitro–ex vivo–in vivo) to assess the actual biological impact of polyphenols on platelet function. Moreover, the study by Ostertag et al. [98] indicated that the concentrations of many phenolic acids that show significant antiplatelet activity are high and non-physiological, whereas relatively lower physiological concentrations demonstrate a nonsignificant effect. Furthermore, non-physiological concentrations of polyphenols used in in vitro analysis questions its bioavailability and/or bioaccessibility [99,100]. An earlier study by Scalbert and Williamson [101] indicated that plasma concentrations of polyphenols are usually between 0.1 µM and 1 µM. However, as reviewed by D’Archivio et al. [50], several factors can affect bioavailability such as food processing, the food matrix, host-related factors, and the type of polyphenol in the diet. Moreover, a review of 97 bioavailability studies showed that plasma concentrations and urinary excretion differ depending on the type and not the proportion of polyphenols in the diet. Due to such important parameters associated with bioavailability, it has been suggested that the metabolites of polyphenols should rather be used to study antiplatelet effects [52,58,102].

Some dietary flavonoids and their metabolites have exhibited significant antiplatelet effects when used in synergy with aspirin [103]. Thus, it may be inferred that administering dietary polyphenols in conjunction with antiplatelet drugs may enhance therapeutic effects. However, polyphenols exhibit paradoxical effects. Apart from the liver and intestinal biotransformation that polyphenols undergo during metabolism, they can suppress the activity of cytochrome P450 enzymes found in both organ sites [104,105]. Since these enzymes are involved in drug metabolism, suppression of their activity can lead to unfavourable high doses of the drug in circulation. Hence, although polyphenols may possess antiplatelet properties their coadministration may not be safe. Further investigations into possible polyphenol-drug interactions are warranted to address these issues. Moreover, although antiplatelet activity has been strongly linked to antioxidant properties of polyphenols, prooxidant effects have also been shown in vitro [106,107]. These prooxidant effects have been reported mostly in cell culture studies with little relation to platelet activation studies [106]. The low bioavailability of polyphenols has led to the speculation that prooxidant or antioxidant activity is not significant in vivo. However, evidence of several dietary intervention studies and epidemiological data strongly link dietary polyphenol consumption (with high antioxidant activity) to the alleviation of oxidative stress-induced platelet activation [98,108].

Although dietary intervention trials have been used to try to address the limitation of in vitro investigations, the use of larger sample sizes and well-controlled studies is needed to reduce the risk of bias. According to the review by Marx et al. [109], which systematically evaluated a limited number of polyphenol-rich intervention studies with low-risk bias, polyphenols may improve cardiovascular disease markers. This review, however, did not take into account studies investigating the antiplatelet effects of polyphenols, as seen in the oat intervention study by Zhang et al. [75]. In this study, avenanthramides may not be the sole compounds responsible for the antiplatelet effects. Although known to have high bioavailability in humans, avenanthramides may be acting synergistically with nutrients other than polyphenols to modulate antiplatelet effects [78]. The same may apply with other dietary intervention studies and thus care should be taken in interpreting and drawing practical implications. Detailed knowledge of the composition of polyphenol-rich extracts and diets may help in deducing the structure–activity relationship. Another variable essential to addressing the hypothesis in all these studies is the type of assay used.

Two common types of assays used in these studies are aggregometry and flow cytometry. A fundamental difference between both methods is that whereas aggregometry measures the extent of platelet aggregation upon activation, flow cytometry is more sensitive in that it detects the expression of activation-dependent surface markers on platelets. Upon comparing different platelet aggregation tests between each other and the flow cytometry, Gremmel et al. [7] discovered that the strength of the correlation was dependent on the agonist used. ADP-induced aggregation correlated significantly between aggregometry tests (LTA, VerifyNow and Multiple Electrode Aggregometry) and also translated partly into flow cytometry measurement of P-selectin and GPIIb/IIIa. Poor correlations were observed using arachidonic acid as an agonist. Furthermore, each test has its benefits and drawbacks. Flow cytometry, for example, is more sensitive than aggregometry but does not consider shear stress, hence is a poor simulation of in vivo processes. However, a shear stress-based test like PFA is less sensitive in detecting an ADP receptor antagonist effect [10]. This highlights the importance of adopting a combination of the different types of assays to gain a broader picture of the antiplatelet effects of polyphenols.

Ideally, for a better comparison of studies, a panel of different agonists could be used. Like the study by Gremmel et al. [7], a study investigating the correlation between platelet aggregation and flow cytometry-based platelet activation and PMP generation could be worthwhile. However, a limitation to a study like this may be the poor standardization of PMP measurements. Cointe et al. [110] tried to standardize PMP enumeration across 44 different labs with different flow cytometers. Results indicated a real potential for standardizing PMP enumeration using Megamix standardization beads. In addition, standardization of agonist-induced PMP generation may aid to compare PMP enumeration with platelet activation. Recabarren-Leiva and Alarcón [111] recently attempted to standardize agonist-induced activation while using an antiplatelet agent, aspirin, to evaluate inhibition of PMP generation. Following this investigation, efforts to standardize agonist-induced PMP generation within or across labs may be useful to study potential polyphenol antiplatelet activity. Another variable that is essential in addressing the hypothesis is the population being studied.

Variability in results from polyphenol-platelet studies may arise from the study group. The review by Ostertag et al. [58] suggested that the health status of the study population does not seem to affect the platelet activation result. However, it has been shown, especially in aggregometry assays, that factors such as gender and race in healthy subjects may affect platelet function results [45]. A study aimed at evaluating the reference intervals of flow cytometry-based platelet function results found that ADP-induced platelet activation was significantly higher in females than males [112]. On the contrary, some studies have demonstrated no significant antiplatelet effect in hypertensive populations [93,94,96] (Table 1). Chokeberries and grapes used in these intervention studies are food sources that have previously shown antiplatelet effects in vitro [43,113]. This may highlight that biological variables and different baseline platelet functions of study populations may lead to differences in the antiplatelet effect of polyphenols.

## 6. Conclusions and Future Perspective

In general, there is strong evidence from the literature of the antiplatelet effects of dietary polyphenols. Polyphenols from both aggregometry- and flow cytometry-based studies have displayed the potential to inhibit many aspects of platelet function including the release of platelet microparticles. However, a combination of both platelet function tests, together with specialized methods such as ELISA and electron microscopy, may elucidate details of the antiplatelet effects. Furthermore, the standardization of aggregometry and flow cytometry assays will allow better comparison of studies and the use of platelet microparticles as a biomarker to assess antiplatelet activity of polyphenols.

Despite the bioavailability limitations, there is evidence from interventional trials that consumption of dietary polyphenols may improve cardiovascular health by virtue of their antiplatelet properties. However, the gaps in the literature and limitations of previous studies need to be addressed. Well-controlled bioavailability studies on dietary polyphenol food sources will be useful in developing an understanding of any potential antiplatelet effects of polyphenol-rich diets.

## Figures and Tables

**Table 1:** Effect of polyphenols on platelet activity.

Design Model	Polyphenol/Source	Population	Study Description	Aggregometry (Agg) / Flow Cytometry (Flow)	Findings	Reference
In vitro	Grape seed extract	30 healthy subjects	ADP-induced aggregation and VASP phosphorylation to evaluate inhibition of GSE (7.5 µg mL^−1^ and 15 µg mL^−1^)	Both	Reduced ADP-induced platelet aggregation and platelet reactivity index (by increasing VASP phosphorylation)	[43]
In vitro	Olive leaves	11 healthy male subjects	Randomized single blind study. Evaluation of inhibition of platelet aggregation and ATP release by olive leaf extract polyphenols	Agg	Significant dose-dependent inhibition of aggregation and ATP release	[59]
In vitro	Grape seed extract	5 healthy subjects	TRAP and thrombin-induced activation of gel filtered platelets to compare antiplatelet property of GSE (1.25–50 µg mL^−1^) with pure resveratrol	Both	Inhibition of TRAP and thrombin-induced P-selectin expression, PMP formation, platelet aggregation, and superoxide anion radicals generated from platelets in a dose-dependent manner	[81]
In vitro	Propolis	10 healthy subjects	ADP-induced platelet aggregation to evaluate inhibition 21 propolis polyphenolic extracts	Agg	Most potent inhibitor of aggregation contained luteolin, apigenin, and chrysin	[71]
In vitro	Extracts of the Arnica montana L. flower head and walnut husks (Juglans regia L)	55 healthy subjects	Inhibition of A. montana or J. regia polyphenol extracts (7.5 and 15 µg mL^−1^) using: (a) Arachidonic acid-, collagen-, and ADP-induced aggregation. (b) VASP phosphorylation and platelet surface marker expression.	Both	Significant inhibition of ADP-induced aggregation but not collagen or arachidonic acid. No significant effect on P-selectin or GPIIb/IIIa expression. Significant decrease in VASP phosphorylation	[42]
In vitro	Virgin olive oil polyphenols	6 healthy subjects	Single-blind study. Anti-aggregation effect of hydroxytyrosol acetate (HT-AC) compared with hydroxytyrosol (HT) and aspirin in whole blood and PRP	Agg	HT-AC anti-aggregation effect higher than HT but similar to aspirin	[70]
In vitro and in vivo	Chlorogenic acid	6 healthy subjects (18 mice also used for in vivo assays)	Activation of washed platelets by multiple agonist to evaluate chlorogenic acid (0.1 to 1 mmoL/L) effect on aggregation, secretion, platelet surface marker expression, cAMP levels, and PKA activation. Chlorogenic acid inhibition of thrombus formation assessed in vivo	Both	Dose dependent inhibition of ADP-, collagen-, arachidonic acid-, and TRAP-6-induced aggregation, ATP release P-selectin expression, and GP IIb/IIIa expression. Increased intraplatelet cAMP and PKA activation. Inhibition of in vivo thrombus formation	[61]
In vivo	Anthocyanin C3G from purified black rice	60 male mice	Mice randomly allocated to one of the 3 groups with 20 subjects for each group: control group (normal diet), high-fat diet (HFD group), or an HFD supplemented with C3G	Both	Decreased platelet activation, serum lipid levels, and inhibits platelet ATP release	[63]
In vivo	Red wine polyphenols (Provinols™)	149 (uniephrectomized male Sprague-Dawley rats)	Rats were randomly grouped based on being treated with or without aldosterone-salt, with or without Provinols (20 mg/kg/day) or spironolactone (30 mg/kg/day) for 4 weeks	Flow (including erythroaggregometer using in vitro shear stress-induced platelet activation)	Provinols decreased circulating microparticles independent of shear stress or mineralocorticoid receptor activation	[84]
In vivo	Cocoa	16 healthy male subjects	Double-blind, crossover study. Placebo-controlled. Eight trained and untrained subjects randomly assigned to receive placebo or cocoa polyphenol supplements (236 mg/day) over a week and then afterwards subjected to one hour of exercise	Both	No change in collagen induced aggregation post exercise. ATP release higher post exercise in both trained and untrained groups. Cocoa supplementation administered over a week did not normalize platelet activity after exercise	[56]
In vivo	Chicory coffee	27 healthy subjects	300 mL chicory coffee every day for 1 week	Agg	Variable effects on platelet aggregation depending on the agonists used	[91]
In vivo	High polyphenol beverage	103 healthy athletes	Randomized, double-blind study. Group 1 received a polyphenol-rich beverage, Group 2 a placebo. Samples were collected three weeks before, one day before, immediately, as well as 24 h and 72 h, after a marathon run	Agg	Control group demonstrated a 2.2-fold increase in platelet aggregation after marathon completion. No increase in platelet aggregation in polyphenol-rich beverage group	[92]
In vivo	Polyphenol-rich grape wine	60 untreated, mildly hypertensive subjects	Double-blind placebo-controlled crossover study. Grape juice extract; grape and wine extract each for 4 weeks including a 2-week run-in period	Agg	No effect on ADP, collagen, or epinephrine induced platelet aggregation	[93]
In vivo	Polyphenol-rich grape seed extract	35 untreated subjects with pre and stage 1 hypertension	Double-blind, placebo-controlled, randomized, parallel-group intervention with 300 mg/day grape seed extract capsule. Eight-week intervention period.	Agg	Did not affect platelet aggregation	[94]
In vivo	Flavanol-rich chocolate	20 patients with congestive heart failure	Double-blind, randomized placebo-controlled trial. Supplementation – (2 h after ingestion of a chocolate bar) and long term (4 weeks, two chocolate bars/day).	Agg	Platelet adhesion significantly decreased 2 h after flavanol-rich chocolate ingestion. No effect post 2- and 4-week supplementation	[95]
In vivo	Chokeberry (Aronia mitschurinii) products	38 patients with untreated mild hypertension	16-week single blinded crossover trial. Cold-pressed 100% chokeberry juice (300 mL/d) and oven-dried chokeberry powder (3 g/d), or placebo for 8 weeks without washout.	Agg	No change in platelet aggregation	[96]
In vivo	Oats	22 type 2 diabetes subjects	Randomized crossover involving 8-week intervention with either oat enriched diet (OAT) or a standard dietary (SDA) advice diet. Preintervention habitual (HAB) intakes were used to compare responses.	Flow	Decrease in tissue factor-activated platelets (CD142) after OAT than HAB or SDA. Decrease in tissue factor positive PMPs and fibrinogen-positive PMPs with OAT intervention.	[75]
In vivo	Anthocyanin rich beverage	21 sedentary subjects	Double-blind placebo controlled. Queen garnet plum juice (200 mL/day) were consumed for 28 days	Both	Reduced ADP, collagen, and arachidonic acid-induced platelet aggregation. Reduced P-selectin expression	[97]

ADP, adenosine diphosphate; ATP, adenosine triphosphate; C3G, cyaniding-3-glucoside; GSE, grape seed extract; PKA, protein kinase A; TRAP, thrombin receptor-activating peptide; VASP, vasodilator-stimulated phosphoprotein.

## Abbreviations

ADP	Adenosine diphosphate
ATP	Adenosine triphosphate
C3G	Cyanidin-3-glucoside
cAMP	Cyclic adenosine monophosphate
COX-1	Cyclooxygenase-1
DAG	Diacylglycerol
GSE	Grape Seed Extract
NO	Nitric oxide
PAR	Protease-activated receptor
PIP2	Phosphatidylinositol 4,5-bisphosphate
PI3K	Phosphatidylinositide-3-kinase
PKC	Protein kinase C
PLC	Phospholipase C
PMP	Platelet microparticle
PPP	Platelet poor plasma
PRP	Platelet rich plasma
TRAP	Thrombin receptor-activating peptide
TxA2	Thromboxane A2
vWF	Von-Willebrand factor
5-HT	5-hydroxytryptamine
VASP	Vasodilator-stimulated phosphoprotein
